# Sulforaphane enhances irradiation effects in terms of perturbed cell cycle progression and increased DNA damage in pancreatic cancer cells

**DOI:** 10.1371/journal.pone.0180940

**Published:** 2017-07-10

**Authors:** Patrick Naumann, Jakob Liermann, Franco Fortunato, Thomas E. Schmid, Klaus-Josef Weber, Jürgen Debus, Stephanie E. Combs

**Affiliations:** 1 Department of Radiation Oncology, University Clinic Heidelberg, Heidelberg, Germany; 2 Department of General, Visceral and Transplantation Surgery, University Clinic Heidelberg, Heidelberg, Germany; 3 Department of Radiation Oncology, Klinikum rechts der Isar, Technical University Munich, Munich, Germany; 4 Institute of Innovative Radiotherapy (iRT), Department of Radiation Sciences (DRS), Helmholtz Zentrum München, Munich, Germany; University of South Alabama Mitchell Cancer Institute, UNITED STATES

## Abstract

**Background:**

Sulforaphane (SFN), an herbal isothiocyanate enriched in cruciferous vegetables like broccoli and cauliflower, has gained popularity for its antitumor effects in cell lines such as pancreatic cancer. Antiproliferative as well as radiosensitizing properties were reported for head and neck cancer but little is known about its effects in pancreatic cancer cells in combination with irradiation (RT).

**Methods:**

In four established pancreatic cancer cell lines we investigated clonogenic survival, analyzed cell cycle distribution and compared DNA damage via flow cytometry and western blot after treatment with SFN and RT.

**Results:**

Both SFN and RT show a strong and dose dependent survival reduction in clonogenic assays, an induction of a G2/M cell cycle arrest and an increase in γH2AX protein level indicating DNA damage. Effects were more pronounced in combined treatment and both cell cycle perturbation and DNA damage persisted for a longer period than after SFN or RT alone. Moreover, SFN induced a loss of DNA repair proteins Ku 70, Ku 80 and XRCC4.

**Conclusion:**

Our results suggest that combination of SFN and RT exerts a more distinct DNA damage and growth inhibition than each treatment alone. SFN seems to be a viable option to improve treatment efficacy of chemoradiation with hopefully higher rates of secondary resectability after neoadjuvant treatment for pancreatic cancer.

## Introduction

Despite its low incidence pancreatic cancer is still the fourth leading cause of cancer death. While progress and innovation in oncology managed to improve 5-year survival rates of all tumor entities by approximately 20%, advances have been slow for pancreatic cancer [1]. Main reasons are lack of early symptoms with subsequent late diagnosis mostly at advanced or even metastasized stages as well as a relative resistance to chemotherapeutics and irradiation (RT) [2]. Hence, until today surgical resection is the only realistic chance for cancer cure. Unfortunately, at time of diagnosis less than one fourth of all patients have a disease that is amenable to surgical resection. Therefore, neoadjuvant treatment concepts are still in demand [3, 4]. In locally advanced stage, which comprises none metastasized but due to vessel involvement inoperable tumors, neoadjuvant chemoradiation is a reasonable treatment choice to potentially reach secondary resectability that improves survival rates significantly [3, 5, 6].

Cytotoxic effects of RT are mediated by damage to the DNA such as single or double strand breaks (SSB or DSB) where the latter is less frequent but correlates most with cell killing [7]. In mammalian cells the harmful DSBs are mainly repaired by nonhomologous end joining (NHEJ) [8, 9]. The alternative repair mechanism of homologous recombination (HR) is well conserved in pro- as well as eukaryotes but was reported to be low in pancreatic cancer cells [10]. Usually quickly after occurrence of a DSB the histone H2A that is a part of the DNA-stabilizing nucleosome becomes phosphorylated. This phosphorylation to its γH2AX form is crucial for marking the damage and subsequent recruiting of DNA repair proteins [11]. As a first step in NHEJ the Ku heterodimer (Ku 70 and 80) binds to the broken DNA ends and interacts with further proteins such as X-ray repair cross-complementing protein 4 (XRCC4) that process repairing [12].

Sulforaphane (SFN) is an herbal isothiocyanate that typically occurs in cruciferous vegetables like broccoli and cauliflower. In recent years, it gained scientific popularity for its cancer preventive attributes as well as its antitumor effects. SFN-induced tumor cell growth suppression in pancreatic cancer was reported to be linked with Sonic hedgehog signaling, interaction with Hsp90, oxidative stress, induction of macroautophagy and inhibition of histone deacetylases (HDAC), enzymes that are expressed aberrantly in pancreatic cancer cells [13–16]. Furthermore, SFN can counteract dysregulation in gap junctional intercellular communication, a typical phenomenon in aggressive pancreatic cancer [17]. For head and neck cancer cells radiosensitizing properties were reported after SFN exposition but in pancreatic cancer knowledge about combination of SFN and RT is scarce [18].

In the present study, we sought to assess the role of SFN in combination with photon RT in pancreatic cancer cells *in vitro*.

## Material and methods

### Cell culture

Human pancreatic cancer cell lines AsPC-1, BxPC-3, MIA PaCa-2 and Panc-1 were obtained from CLS Cell lines or ATCC and grown under standard conditions in cell culture flasks according to manufacturer’s instruction. Media were supplemented with 1% (v/v) glutamine-streptomycin solution (Thermo Fisher Scientific Inc., Waltham/Massachusetts) and 10% (v/v) fetal bovine serum (Biochrom GmbH, Berlin). Cells were passaged using EDTA when having formed layers of 70–80% confluence, diluted appropriately and plated in new tissue culture flasks with fresh medium.

### Irradiation

Radiotherapy was performed with photons as single dose at room temperature using our institutional radiobiological X-ray device (XRAD 320 Precision X-Ray, North Branford, Connecticut, USA). Photons were delivered at 320 kV, 12.5 mA with a filter consisting of 1.5mm Al, 0.25mm Cu and 0.75mm Sn. The averaged dose rate was 1 Gy per minute. After irradiation culture flasks were incubated again under standard conditions.

### Clonogenic assays

Equal amounts of cells were plated in culture flasks and incubated in fresh medium for 24 h. Flasks were then treated in triplicates by DMSO or the phytotherapeutic agent SFN (Sigma-Aldrich, Germany). After 24 h the media of all flasks were renewed to stop chemotherapeutic exposure. Cells scheduled for RT were irradiated in triplicates. Finally all flasks were incubated for 8–9 days. Afterwards the remaining cells were fixated with 70% ethanol and stained with trypan blue (Sigma-Aldrich, Germany). In order to determine cell survival all cell clusters of at least 50 cells were counted in every flask and resulting mean values of the triplicates were divided by plated cells. This plating efficiency was normalized to the plating efficiency value of the mock treated negative control. Cell survival was finally expressed as mean of three independent experiments ± SEM.

In order to rate the efficacy of combined treatments their survival was compared to a theoretical control curve with assumed isoeffective radiation doses [19]. If the survival rate was plotted below this theoretical curve the combined treatment could be considered supra-additive and in contrast radioprotective if it was plotted above the curve of mock treatments. Additive effects were concluded if the survival curve was between the theoretical curve and the curve of the negative control, within the so called “window of additivity” [19]. Curves were generated with Sigma Plot (Systat Software, San Jose, California, USA).

Moreover, the impact of co-treatment was assessed by calculation of individual sensitizer enhancement ratios (SER). A SER is defined as the quotient of the radiation dose without sensitizer and of that in the presence of the sensitizer where each radiation dose results in the same clonogenic survival rates *x*.

$$SER=\frac{D_{1}(\mathrm{survival}\;x\;\mathrm{without}\;\mathrm{sensitizer})}{D_{2}(\mathrm{survival}\;x\;\mathrm{with}\;\mathrm{sensitizer})}$$ (1)

The radiation doses where estimated by linear quadratic regression derived from clonogenic survival results and SERs calculated for isoeffective surviving fractions x of 50%, 10% and 1% for each cell line and SFN concentration if applicable.

### Cell cycle assays

Cells were equally seeded in culture flasks and incubated for 22 h. One probe was fixated as baseline time point (-2 h) and others were treated with DMSO or SFN. After 2 h of drug exposure the media of all cells were renewed and probes scheduled for RT were irradiated. At this 0 h time point as well as 12 h and 24 h later a probe of each treatment scheme was fixated. For fixation cells were first detached using EDTA, then re-exposed to fresh media and centrifuged. The resulting cell pellet was washed with cold PBS and finally resuspended in ice cold ethanol. Before cell cycle analysis cells were again centrifuged, the supernatant was discarded and the cell pellet resolved in PBS. This process was repeated twice in order to wash out ethanol residues. The PBS cell solution was then incubated for 10 min with RNAse to prevent RNA-interactions with DNA-stain propidium iodide that was added afterwards. Finally, cell cycle analysis was conducted at a flow cytometer (FACScan™ Becton & Dickinson, Franklin Lakes, New Jersey, USA) with the associated software BD CellQuest™. The distribution of cell cycle phases G1, S and G2 were then determined with ModFit LT (Verity Software, Topsham, Maine, USA).

### Pan-nuclear γH2AX quantification

Cells were seeded equally in Petri dishes, incubated for 24 h and then treated with DMSO or SFN for 24 h. After medium change of all probes some dishes were exposed to RT. Cells were then detached from the dishes 1 h and 12 h after medium change using EDTA and resuspended in fresh medium, centrifuged, washed with PBS and finally fixated with ethanol. For quantification of γH2AX cells were again washed with PBS to remove ethanol, then permeabilized with Triton-X and incubated with γH2AX antibody (Merck KGaA, Darmstadt, Germany). Data analysis was performed with laser scanned flow cytometry as described previously [20].

### Western blot

Cell lines were seeded equally in Petri dishes, incubated for 24 h and afterwards exposed to DMSO, SFN and/or RT. After 24 h incubation the media were renewed and cells were harvested gently, incubated in lysis buffer and equal amounts of protein homogenates were loaded to a 12% SDS-PAGE gel. After electrophoretical separation the proteins were transferred from the gel to a nylon membrane as described previously [15]. Membranes were then incubated with the primary anti-Ku70, anti-Ku80 (each Cell Signaling Technology Danvers, USA) or anti-XRCC4 (Santa Cruz, Dallas, USA) followed by incubation with HRP-conjugated secondary anti-mouse or anti-rabbit antibody (Santa Cruz, Dallas, USA). Each membrane was stripped with Western blot stripping buffer and re-probed with GAPDH (Cell Signaling Technology Danvers, USA) in order to confirm equal protein loading. Quantification of band density was done using the open source image processing software ImageJ and its Gel Analysis routines. Each target protein density was normalized to the density of its loading control value (GAPDH).

### Statistical analysis

Statistical analysis was performed using Student’s *t-Test* for each experimental group. Results were considered significantly different when the obtained *p* value was less than 0.05. Statistical calculations were performed and graphs generated with Prism software (GraphPad, USA).

## Results

### Exposure to radiation and SFN strongly inhibits cellular growth

**Fig 1:**
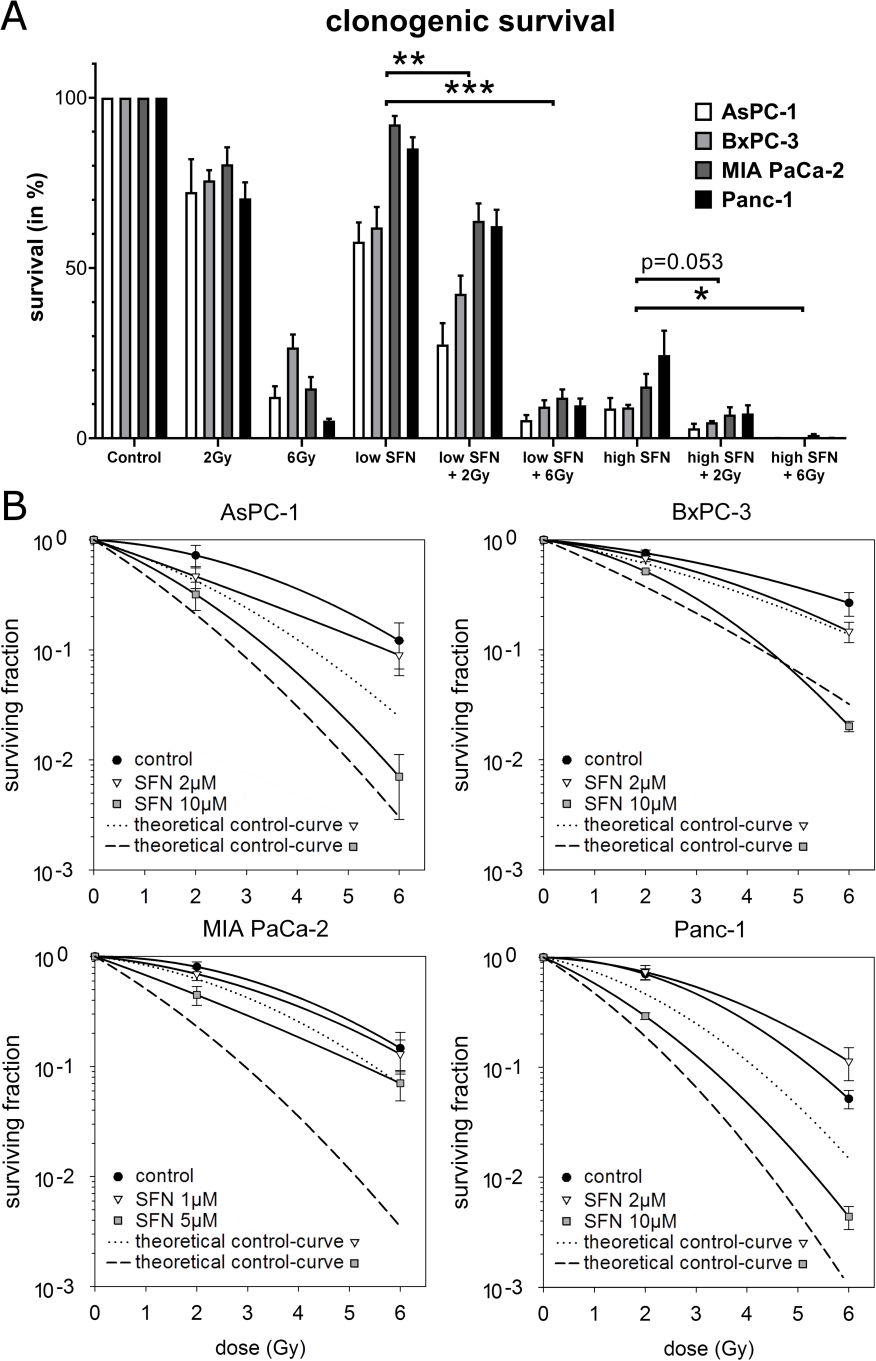
Clonogenic survival results. Results of clonogenic survival experiments (n = 3) for each pancreatic cancer cell line after exposure to SFN in a low (2 μM, for MIA PaCa-2 only 1 μM) or high dose (10 μM, for MIA PaCa-2 only 5 μM) for 24 h followed by RT in a low (2 Gy) or high dose (6 Gy), and combination of both. (A) Survival grouped by treatments including Students t-test statistics (* p<0.05, ** p<0.01, *** p<0.001, **** p<0.0001). (B) Logarithmic survival as function of dose for radiation and co-treatments as well as calculated artificial theoretical control curves of assumed isoeffective radiation doses for low (dotted) and high dose SFN (dashed), respectively. Co-treatments with a surviving fraction below the respective theoretical control curve can be considered as supra-additive (see materials and methods section).

To determine whether efficacy of radiotherapy (RT) could be enhanced by sulforaphane (SFN) we exposed the four established and exponential growing pancreatic cancer cell lines AsPC-1, BxPC-3, MIA PaCa-2 and Panc-1 to SFN and/or RT. Treatment efficacy was assessed by clonogenic survival (Fig 1A). An exposure of 2 Gy or 6 Gy reduced surviving cell clones of all cell lines highly significant to a mean of 73% (SD 9.7%) and 15% (SD 4.7%), respectively. Single treatment with SFN for 24 h lowered clonogenic survival rates averaged over all cell lines to quite similar values of 74% (SD 7.6%) and 14% (SD 6.4%) for a low and a high dose, respectively. The SFN doses were 2 μM and 10 μM for AsPC-1, BxPC-3 and Panc-1 and only half for MIA PaCa-2 (1 μM and 5 μM) since the latter cells appeared especially sensitive to SFN.

After determination of dose concepts for a low and a high effective single treatment we combined both SFN and RT. Co-treatment resulted in a pronounced reduction of mean clonogenic survival rates to 49% (SD 9.3%) and 9.1% (SD 3.4%) for low dose SFN plus RT, and 5.5% (SD 2.7%) and 0.4% (SD 0.2%) for high dose SFN plus 2 Gy or 6 Gy, respectively. Differences were again dose dependent and highly significant (Fig 1A).

To describe additivity of combined treatments we plotted the logarithmic surviving fraction of each cell line as a function of dose together with a calculated theoretical control curve of assumed isoeffective radiation doses as described in the materials and methods section. This approach allows the discrimination of additive and supra-additive treatment effects [19]. Since all surviving fractions of combined treatments were below the curve of controls (just RT) additive effects of SFN plus RT can be assumed. In BxPC-3 cells the clonogenic survival of RT plus high dose SFN was even below their respective theoretical control-curve indicating supra-additive effects in these cells and suggesting possible SFN-caused radiosensitization (Fig 1B).

Another approach to describe additivity of combined treatments is the calculation of sensitizer enhancement ratios (SER). Such a ratio describes the magnitude by which the RT dose must be multiplied to induce a same estimated surviving fraction in the absence of the sensitizer. We calculated the SERs for an estimated survival of 50%, 10% and 1% for the above mentioned low and high concentration of SFN for each cell line (Table 1). In accordance with the clonogenic survival rates the SERs for AsPC-1 and BxPC-3 were higher than for MIA PaCa-2 and Panc-1.

**Table 1:** Sensitizer enhancement ratios (SER). SERs for an estimated 50%, 10% and 1% surviving fraction for every cell line and each for low and high concentrations of SFN.

SER for an estimated survival	low dose SFN			high dose SFN	
	50%	10%	1%	10%	1%
AsPC-1	5.74	1.56	1.35	n.a.^†^	4.86
BxPC-3	4.68	1.83	1.61	n.a.^†^	5.99
MIA PaCa-2	1.22	1.11	1.09	8.23	2.52
Panc-1	1.36	1.12	1.08	6.92	3.87

^†^ SER is not applicable (n.a.) if SFN treatment alone resulted in less survival than the estimation.

### SFN blocks G2/M cell cycle progression especially when combined with irradiation

**Fig 2:**
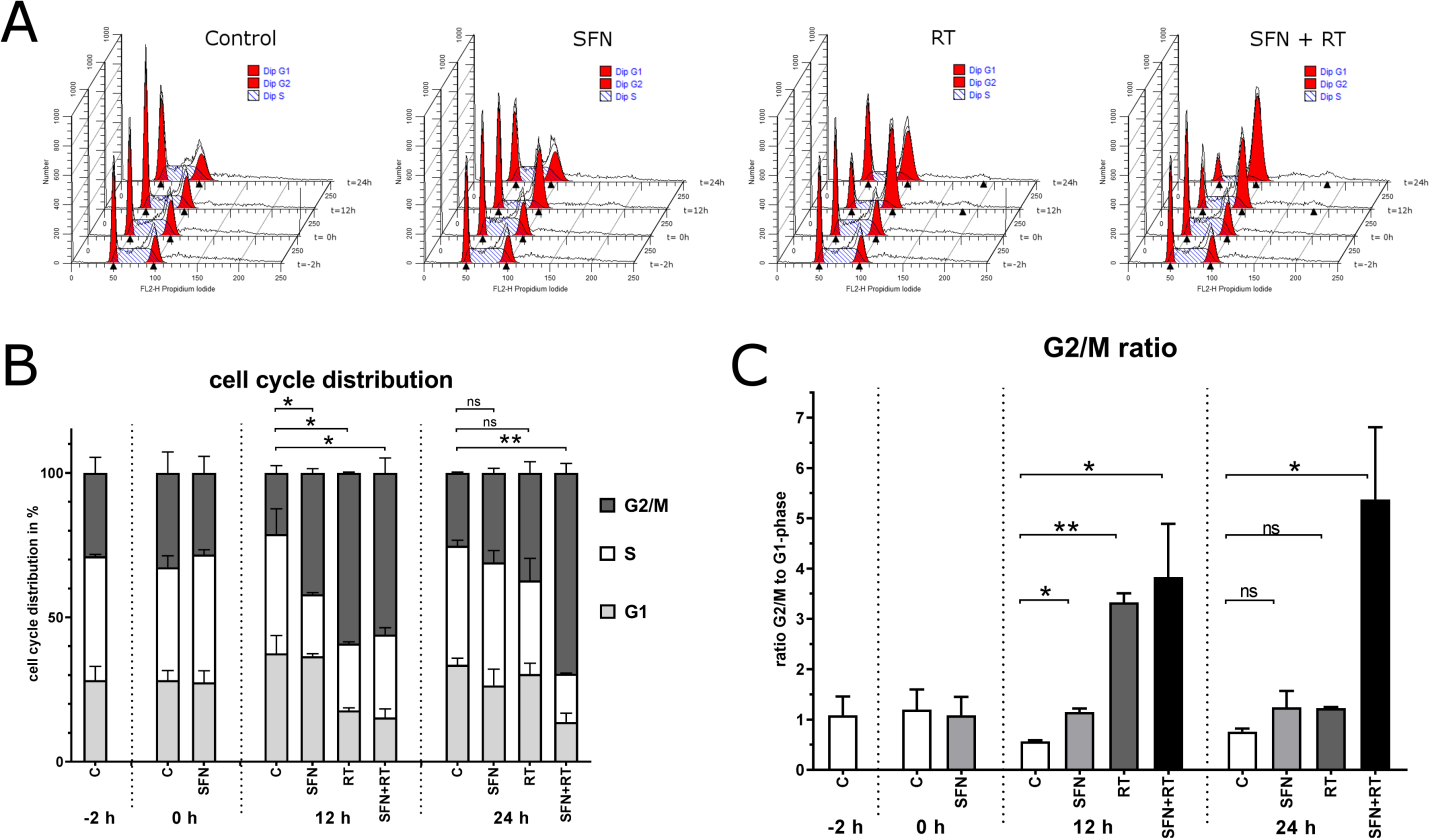
Cell cycle distribution in Panc-1 cell. Cell cycle distribution at different time points: -2 h is the start of 40 μM SFN exposure, 0 h the time point of RT with 4 Gy and the other time points mark 12 h and 24 h after treatment. (A) Example of stacked ModFit curves of Panc-1 cell cycle raw data at the different time points and for the different treatments with G1 and G2 peaks. (B) Relative cell cycle distribution of G1, S and G2/M phase for Panc-1 cells including results of Students t-test of G2/M proportions between control and treatments (* p<0.05, ** p<0.01). (C) Ratios of G2/M and G1 proportion including results of Students t-test in comparison to the respective control (* p<0.05, ** p<0.01).

To asses if growth inhibition by SFN was caused by perturbation in cell cycle progression we performed flow cytometric cell cycle analysis 12 h and 24 h after treatment with SFN and/or irradiation. For both SFN and irradiation we observed a statistically significant accumulation of cells in G2/M phase 12 h after treatment. This G2/M- accumulation occurs mostly at cost of cells in G1 phase but was already in remission 24 h after single treatment with an again nearly reset G1 proportion compared to the control group. In contrast, co-treatment of SFN and irradiation induced a pronounced G2/M-arrest that was even higher at 24 h than after 12 h and still statistically significant (Fig 2A and 2B). This treatment related disturbance of cell cycle progression becomes even clearer by plotting the ratio of cells in G2/M to G1 phase (Fig 2C). At 12 h ratios were significantly higher for single as well as co-treatments whereas at 24 h only the combination of SFN and irradiation still showed a significant aggravated ratio compared to mock treatments.

The described effects were observed in all four cell lines and when overall statistics were calculated (Table 2) there was a highly significant difference of cells in G2/M phase at 12 h for each treatment and after 24 h just for co-treatments. For RT alone test statistics after 24 h also reached significance over all cell lines but averages of G2/M population were distinctive less and the p-value higher than in the SFN + RT group. When statistics were compared with the RT only groups instead of mock treated controls the single treatments with SFN showed significant less G2/M proportions after 12 h and 24 h whereas after 24 h co-treatments led to distinct higher proportions with higher significance, too.

**Table 2:** Statistics of G2/M population over time. Means of relative G2/M population in % (± SEM) with statistics (n = 3; t-test).

time	treatment	AsPC-1	BxPC-3	MIA PaCa-2	Panc-1	p-value vs. control	p-value vs. RT
-2 h	Control	27.6 ±10.7	29.4 ± 5.3	20.9 ± 0.1	28.9 ± 5.4		
0 h	control	29.4 ± 8.7	32.0 ± 0.7	19.6 ± 1.6	32.6 ± 7.3		
	SFN	27.4 ± 10.0	25.4 ± 1.3	16.6 ± 0.7	28.3 ± 5.7	n.s.	
12 h	control	19.3 ± 0.3	28.2 ± 3.5	21.4 ± 2.1	21.1 ± 2.5		
	RT	63.5 ± 0.1	50.8 ± 1.0	59.5 ± 1.3	59.1 ± 0.3	<0.0001	
	SFN	44.8 ± 5.2	34.7 ± 4.1	48.9 ± 2.6	42.1 ± 1.5	<0.0001	<0.01
	SFN + RT	51.5 ± 5.2	49.4 ± 5.4	56.2 ± 2.5	55.9 ± 10.2	<0.0001	n.s.
24 h	control	26.3 ± 3.4	28.9 ± 5.3	20.1 ± 2.0	25.3 ± 0.3		
	RT	45.0 ± 4.8	47.8 ± 7.5	32.8 ± 5.0	37.2 ± 3.9	0.006	
	SFN	22.3 ± 7.4	25.4 ± 1.7	29.3 ± 5.5	31.0 ± 1.6	n.s.	<0.01
	SFN + RT	59.1 ± 4.5	58.2 ± 4.2	67.5 ± 4.6	69.5 ± 3.3	<0.0001	<0.0001

### DNA damage caused by SFN and irradiation

**Fig 3:**
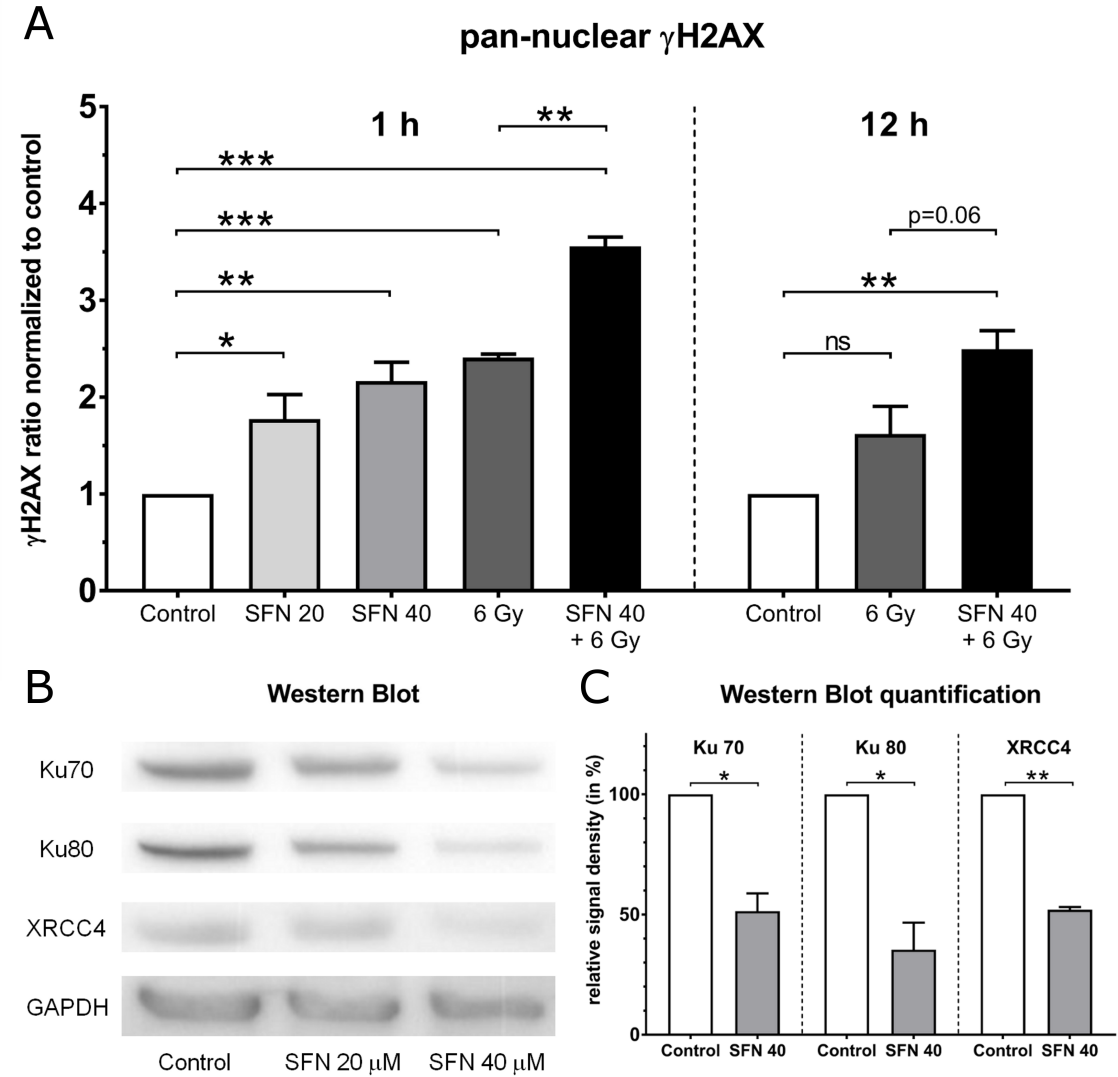
DNA damage in Panc-1 cells. (A) Pan-nuclear γH2AX signals of Panc-1 cells normalized to values of mock treated cells at time points 1 h and 12 h after treatments as surrogates for DNA double strand breaks (n = 3). SFN-treated cells were incubated with either 20 μM or 40 μM SFN for 24 h and afterwards if indicated irradiated with 4 Gy at time point 0 h. (B+C) Western blot of NHEJ pathway proteins after treatment of Panc-1 cells with SFN for 24 h and statistics of their band densities normalized to the loading control. For statistics data of Students t-test are shown (* p<0.05, ** p<0.01, *** p<0.001).

Since effects of RT are mostly explained by damage to DNA we sought to estimate DNA double strand breaks (DNA-DSB) by quantification of pan-nuclear γH2AX. After exposure to SFN we observed a significant and dose dependent accumulation of γH2AX by an average factor of 1.8 and 2.2 for Panc-1 already 1 h after treatment with 20 μM and 40 μM SFN, respectively (Fig 3A). A radiation dose of 6 Gy led to a 2.4-fold increase of γH2AX and co-treatment showed a further increase by a factor of 3.5. This additional γH2AX accumulation was even significant higher than the one caused by irradiation alone. At a later time point, an increase in γH2AX was still measureable after RT alone but reached no statistical significance any more. In contrast, co-treatments showed again a statistical highly significant accumulation of pan-nuclear γH2AX.

Among different cellular responses following DNA-DSBs the gene transcription of DNA repair proteins is altered. Since pancreatic cancer has only low competence in homologous recombination (HR) repair [10] we quantified the protein levels of Ku 70, Ku 80 and XRCC4. These proteins are key players in nonhomologous end joining (NHEJ), the predominant DNA repair response in mammals [9]. After treatment with SFN we observed a dose dependent reduction in protein levels of the Ku heterodimer as well as XRCC4 (Fig 3B). For the higher dose of SFN western blot bands were statistically significant weaker than in DMSO treated mock controls (Fig 3C). Irradiation alone did not show a difference compared to control cells but in combination with SFN we observed similar reduced protein levels as after SFN single treatment (S1 Fig). In fact, co-treatments did not produce any additional effect concerning NHEJ protein levels than SFN alone.

## Discussion

This study presents *in vitro* data showing enhanced antitumor efficacy in four pancreatic cancer cell lines by combining conventional photon irradiation with the phytochemical agent SFN. Treatment efficacy was assessed by clonogenic survival assays as well as flow cytometric tests for cell cycle perturbation and pan-nuclear γH2AX accumulation as surrogate for DNA double strand breaks. Both cell cycle blockade and DNA damage are cornerstones in radiation mediated antitumor effects. Since radiation induced growth inhibition mostly occurs in a longer time scale than cytotoxic chemotherapy, clonogenic assays still represent the traditional gold standard [21]. In all our four established pancreatic cancer cell lines AsPC-1, BxPC-3, MIA PaCa-2 and Panc-1 we observed that treatment with SFN and/or exposition to irradiation leads to a dose dependent and highly significant reduction of clonogenic survival especially in combined treatment. SFN is known as potent inducer of apoptosis as well as macroautophagy in pancreatic cancer cells [15, 22]. Its effects in humans are currently investigated in a clinical phase I study (POUDER-trial) for patients with metastasized pancreatic cancer [23]. Efficacy of RT related cytotoxicity in pancreatic cancer can be enhanced by combination with chemotherapeutics such as gemcitabine [24, 25]. As a matter of fact, chemoradiation as neoadjuvant treatment concept is–if in concern of patient’s general condition reasonable administrable—still more a treatment of choice than RT alone [4, 26]. Combination of SFN and subsequent irradiation in head and neck cancer cell lines inhibited cell proliferation more than each treatment alone [18]. In murine osteosarcoma cells combination of SFN and irradiation has been described to be superior to single treatments concerning growth inhibition, cell cycle perturbation and apoptosis induction [27].

Here we show enhanced efficacy by co-treatment with SFN and irradiation in human pancreatic cancer cells. In all our cell lines additivity was shown according to the model of Steel and Peckham [19]. Supra-additive effects were marginally reached for BxPC-3 cells for high dose SFN with high dose irradiation. But we think that this close and single observation does not allow describing a SFN-caused radiosensitization. In addition, calculated SERs for different survival rates showed always values of greater one indicating at least additive effects.

Cell cycle progression was perturbed in all our cell lines after SFN and/or irradiation with an accumulation of cells in G2/M phase that only after co-treatments persisted at 24 h indicating more severe cellular effects in combined treatment approaches. For MIA PaCa-2 and Panc-1 a similar G2/M-arrest was described in line with growth suppression and apoptosis after treatment with doses of SFN between 2 μM and 40 μM [28]. Photon irradiation in doses of 2 Gy to 8 Gy alone exerts a distinct G2/M blockage in Panc-1 cells among other cell lines [24]. But we could not observe a distinct sub G1 peak that is deemed typically for apoptotic cell death and was for example shown for SFN in the human prostate cancer cell line PC-3 [29]. Maybe our time points with a maximum of 24 h were not long enough to develop a G1 peak. On the other hand, in human ovarian cancer cells a clear G2/M blockade was reported when treated with 12.5 μM SFN up to 72 h whereas a sub G1 peak was also not evident [30].

By quantification of γH2AX, a sensitive indicator protein for DNA double strand breaks [31], we revealed that SFN as well as irradiation alone do disturb DNA stability. In combined treatment, this effect was even more pronounced and lasted over a longer period. Similar results were very recently published for a biolipid purified from spinach namely monogalactosyl diacylglycerol (MGDG) that in combination with 8 Gy irradiation leads to an increase in γH2AX levels [32]. Interestingly, the authors also show an induced G2/M arrest without sub G1 peaks although apoptosis induction was shown separately by DNA fragmentation, cytochrome c release and caspase-3 cleavage.

DNA DSBs can be repaired by either HR or NHEJ pathways. In pancreatic cancer HR competence was reported to be low and in mammals NHEJ is considered the predominant DSB repair mechanism [9, 10]. As a first step in NHEJ the Ku heterodimer binds to the broken DNA ends and then recruits further proteins like DNA-dependent protein kinase (DNA-PK), Artemis, DNA ligase IV and XRCC4 [12, 33]. After irradiation we could not determine differences in protein levels of the Ku heterodimer as well as XRCC4. But our cells showed a dose dependent decrease in NHEJ protein levels after treatment with SFN. Similar results were observed in the rat pancreatic acinar cell line ARJ42 after exposure to oxidative stress [34]. Together with our previously published work where we have shown that SFN induced cell death and macroautophagy induction depend on oxidative stress [15] our current data suggest that SFN directly or via oxidative stress indirectly induces a loss of NHEJ proteins. Moreover, impairment of NHEJ could be an explanation for our observation that combined treatments showed a more sustained cell cycle arrest and slower DNA DSB repair than each treatment alone.

## Conclusion

In conclusion, SFN and RT cause growth inhibition by cell cycle perturbation and DNA damage. Combined treatment had more pronounced effects and lasted over longer periods than each treatment alone. Our data provide good *in vitro* evidence to infer that co-treatment with SFN may enhance effects of RT in pancreatic cancer.

## Supporting information

S1 Fig Western blot of NHEJ proteins. Western blot of NHEJ proteins Ku 70, Ku 80 and XRCC4 after treatment of Panc-1 cells with 2 or 6 Gy RT and/or treatment with either 20 μM or 40 μM SFN for 24 h. GAPDH bands to show equal protein load.
(TIF)
