# Supplementary figures and images for: Sulforaphane enhances irradiation effects in terms of perturbed cell cycle progression and increased DNA damage in pancreatic cancer cells
Source: PLoS One. 2017 Jul 10;12(7):e0180940. doi: 10.1371/journal.pone.0180940 (PMC5507286; doi:10.1371/journal.pone.0180940)

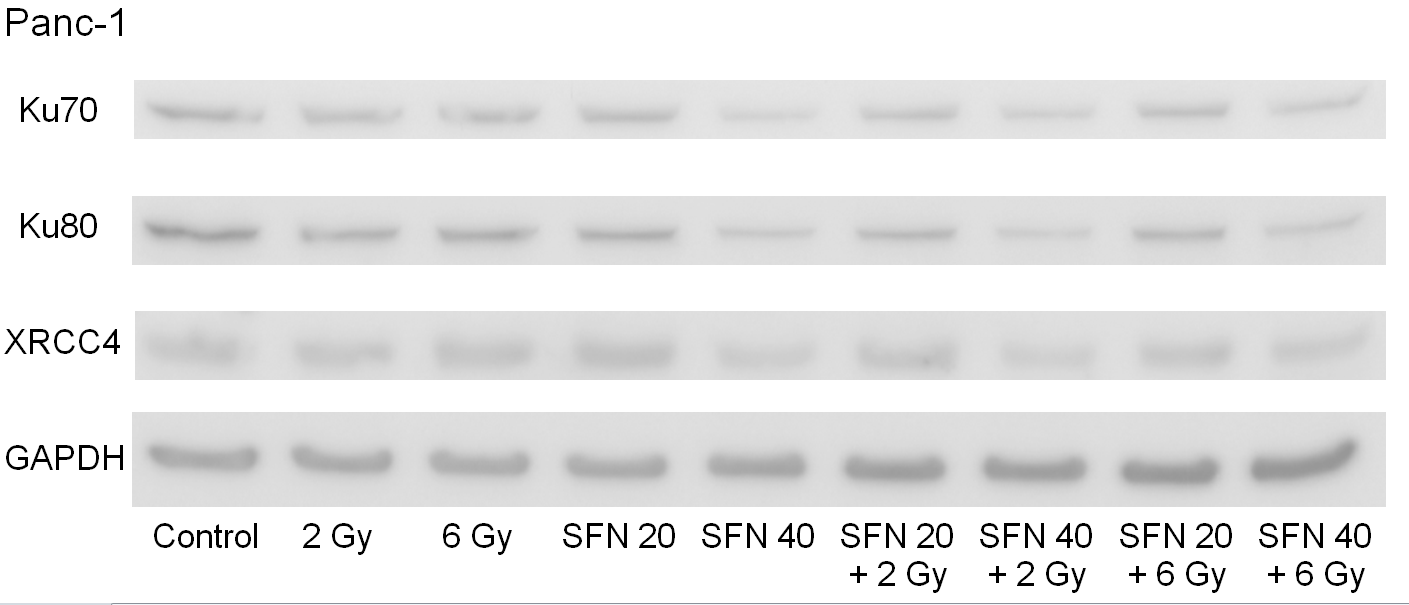

Supplement: S1 Fig — Western blot of NHEJ proteins Ku 70, Ku 80 and XRCC4 after treatment of Panc-1 cells with 2 or 6 Gy RT and/or treatment with either 20 μM or 40 μM SFN for 24 h. GAPDH bands to show equal protein load. (TIF) [file pone.0180940.s001.tif]
